# CO_2_‐mediated bloater defect can be induced by the uncontrolled growth of *Enterobacteriaceae* in cucumber fermentation

**DOI:** 10.1002/fsn3.3557

**Published:** 2023-07-19

**Authors:** Yawen Zhai, Christian G. Pagán‐Medina, Ilenys M. Pérez‐Díaz

**Affiliations:** ^1^ Department of Food, Bioprocessing and Nutrition Sciences North Carolina State University Raleigh North Carolina USA; ^2^ US Department of Agriculture Agricultural Research Service, SEA Food Science and Market Quality and Handling Research Unit Raleigh North Carolina USA

**Keywords:** bloater defect, cucumber fermentation, *Enterobacter*

## Abstract

*Enterobacteriaceae* are known to proliferate in cucumber juice, deriving energy from the fermentation of sugars to organic acids and ethanol, and theoretically generating carbon dioxide (CO_2_). We hypothesized that the CO_2_ produced by the indigenous *Enterobacteriaceae* in the early stage of cucumber fermentation accumulates in the fermenting fruits causing bloater defect. The ability of seven *Enterobacteriaceae*, indigenous to cucumber, to grow and produce CO_2_ in cucumber juice medium (CJM), a sterile model system for cucumber fermentation, was characterized. The induction of bloater defect in cucumber fermentation conducted with pasteurized and acidified fruits was also evaluated. The generation times of the seven *Enterobacteriaceae* in CJM ranged between 0.25 and 8.20 h and resulted in carbon dioxide (CO_2_) production to estimated amounts of 7.22–171.5 mM. *Enterobacter cancerogenus* and *Enterobacter nimipressuralis* were among the bacteria that produced the most and the least CO_2_ in CJM, respectively, at estimated mM concentrations of 171.58 ± 42.96 and 16.85 ± 6.53. Inoculation of *E. cancerogenus* and *E. nimipressuralis* in acidified and pasteurized cucumbers resulted in the production of 138 and 27 mM CO_2_, respectively. Such *Enterobacteriaceae* produced 2% hydrogen in the model cucumber fermentations. A bloater index of 25.4 and 17.4 was calculated from the cucumbers fermented by *E. cancerogenus* and *E. nimipressuralis*, respectively, whereas no defect was observed in the fruits collected from uninoculated control fermentation jars. It is concluded that the metabolic activity of the *Enterobacteriaceae* indigenous to cucumber can produce sufficient CO_2_ in cucumber fermentations to induce bloater defect.

## Graphical abstract

The carbon dioxide formed by *Enterobacteriaceae* in cucumber fermentation is trapped in the fruits causing bloater defect. Understanding that *Enterobacteriaceae* can cause bloater defect propels the development of strategies to prevent economic losses for processors.

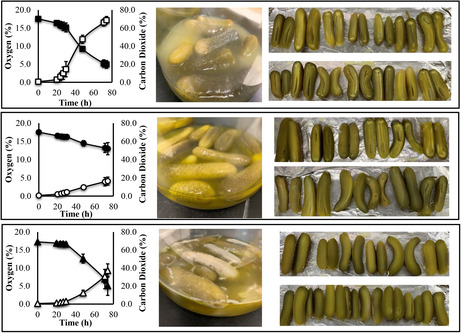

## 1 INTRODUCTION

Bloater defect is commonly observed by the pickling industry in the fermentation of cucumbers (Corey et al., 1983). Cucumbers are fermented in outdoor, open‐top fiberglass tanks and are typically brined with 1.06 M (6%) sodium chloride (NaCl), 15 mM acetic acid, and 25 mM calcium chloride (CaCl_2_) (Pérez‐Díaz et al., 2015). The cover brine ingredients and the fruit water‐soluble components equilibrate in 48–72 h (Passos et al., 2005). The fermentation, led by the indigenous lactic acid bacteria (LAB), proceeds to completion in 21–30 days depending on the ambient temperature. Tissue softening, microbial spoilage, and bloater defect are some of the culprits in the deterioration of fermented cucumber quality. Among these culprits, bloater defect causes serious annual yield and economic losses to the pickling industry impacting between 3% and 40% of the production (Fleming et al., 1973b; personal communication with processors, 2022).

Bloater defect is defined as hollow cavities that form inside the fruits with honeycomb, lens, or balloon shapes (Etchells et al., 1974). A mechanism for the development of bloater defect was proposed in 1980 and refers to the diffusion of gas, mostly carbon dioxide (CO_2_), from the fermentation cover brine to the fruit due to a concentration gradient (Etchells et al., 1968; Fleming et al., 1973a; Fleming & Pharr, 1980).

Production of CO_2_ in cucumber fermentations corresponds to the presence of *Enterobacteriaceae*, such as *Enterobacter* spp., *Leuconostocaceae*, and *Lactobacillaceae* in combination with yeasts (Etchells et al., 1945, 1968; Etchells & Jones, 1941; Fleming et al., 1973a; McDonald et al., 1991; Zhai & Pérez‐Díaz, 2020, 2021). Of such bacterial families, only *Leuconostocaceae* has been directly implicated in the development of fermented cucumber bloater defect (Zhai & Pérez‐Díaz, 2020). The role of *Enterobacteriaceae* and *Lactobacillaceae* as causative agents of bloater defect remains questionable. However, several ɣ‐proteobacteria, including *Enterobacteriaceae*, proliferate in cucumber juice with a generation time of 1.8845 ± 0.4645 h, which is insignificantly different from that of the lactobacilli that prevail in the fermentation of the fruit (Rothwell et al., 2022). Most *Enterobacteriaceae* ferment the intrinsic sugars in CJM to acids (Rothwell et al., 2022). *Enterobacteriaceae* are capable of aerobic and anaerobic growth, and CO_2_, ammonia, and nitrogen production (Iversen, 1994). They are facultative anaerobes that prefer oxygen as an electron acceptor and ferment sugars to produce lactic acid among other acids and CO_2_ (Ciani et al., 2008; Unden & Bongaerts, 1997). Aerobic growth supported by the citric acid cycle is described for *Enterobacteriaceae*, particularly *Enterobacter aerogenes* (Antranikian & Giffhorn, 1987). Specifically, *E. aerogenes* and *Escherichia coli* have been implicated in the production of hydrogen (H_2_) and CO_2_ in commercial fermentations brined with 10% NaCl (Etchells, 1941). It is because *E. aerogenes* and *Es. coli* are known to produce gases in cucumber fermentations brined with 10% NaCl that they have been proposed as contributors to bloater defect (Etchells et al., 1968; Veldhuis & Etchells, 1939). Thus, we aim at elucidating the direct role of *Enterobacteriaceae* in the induction of bloater defect.

This study investigated the ability of seven *Enterobacteriaceae* species autochthonous to cucumber fermentation (Pérez‐Díaz et al., 2018) to produce gases, specifically CO_2_ and hydrogen, to levels that suffice for causing bloater defect in cucumber fermentation. We used CJM as a sterile model system for the fermentation of the fruit to test gas production and growth rate of *Citrobacter freundii*, *E. cancerogenous*, *E. cloacae*, *E. nimipressuralis*, *Pantoea ananatis*, *P. agglomerans*, and *Leclercia adecarboxylata*. Two cultures, *E. cancerogenous* and *E. nimipressuralis*, able to produce the most and the least CO_2_, respectively, in CJM were inoculated in a cucumber fermentation model system to understand their role in the development of bloater defect. Inoculating the *Enterobacteriaceae* in indigenous fermentations would obscure a potential role in the development of bloater defect as the natural cucumber microbiota is diverse in levels and species and could also contribute to the resulting quality of the ferment. The model cucumber fermentation included acidified and pasteurized fruits to eliminate the indigenous cucumber microbiota. The ability of *Enterobacteriaceae* to induce bloater defect was, thus, observed in the absence of microbial competition. It was the objective to define the need for controlling the growth of autochthonous *Enterobacteriaceae* in commercial cucumber fermentation, beyond what acid production and competing microbes do, to prevent bloater defect and minimize economic losses for processors. It was understood that the actual contribution of *Enterobacteriaceae* to bloater defect in indigenous fermentations would differ from that observed in the model system used. However, defining the extent of the contribution of such bacteria to bloater defect would justify and enable the development and implementation of alternative strategies to prevent economic losses.

## 2 MATERIALS AND METHODS

### 2.1 Growth of *Enterobacteriaceae* in CJM

Seven cultures of *Enterobacteriaceae* isolated from days 1 and 3 of industrial cucumber fermentations were used in this experiment including *Citrobacter freundii* 1.2.3E, *Enterobacter cancerogenus* 3.2.13E, *Enterobacter cloacae* 3.2.8E, *Pantoea ananatis* 1.2.16E, *Pantoea agglomerans* 1.2.4E, *Enterobacter nimipressuralis* 1.2.7E, and *Leclercia adecarboxylata* 3.2.10E (Pérez‐Díaz et al., 2018). Such cultures are maintained in the USDA‐ARS Food Science & Market Quality and Handling Research Unit located in Raleigh, North Carolina, USA. The cultures were transferred from frozen stocks to brain–heart infusion (BHI) broth prior to the inoculation of the experimental medium. The BHI cultures were incubated at 30°C under static conditions for 24 h. The inocula were suspended in a 0.85% NaCl solution prior to the inoculation of the experimental media, after centrifugation at 15,294 *g* for 10 min at 22°C (Eppendorf Centrifuge 5810R, Fisher Scientific, Fremont, CA, USA). The inoculum concentration was estimated using McFarland Standards and confirmed by plating on BHI.

CJM was prepared from blended size 3B (1.75–2 in. diameter) fresh cucumbers using a commercial blender assembly for 60 s at maximum speed (Waring Co., Torrington, CT, USA). The blended cucumbers were sieved using cheesecloth and the liquid phase was filtered‐sterilized using a 0.2‐μm filtration unit (Nalgene®‐Rapid Flow™, Thermo Scientific, Santa Clara, CA, USA). One lot of fresh cucumbers was used for this experiment. The sterilized CJM was stored at 4°C until used.

A 96‐well plate format was used for this experiment and growth at 30°C was monitored by measuring absorbance at *λ*_630_ using an ELx808 Absorbance Microplate Reader (BioTek, Winooski, VT, USA). The microplate reader uses a tungsten halogen light source and a photodiode detector to measure absorbance at preset time intervals, which was 1 h for this experiment. Twenty microliters of the inocula of an estimated concentration of 3 log of CFU/mL was added to 180 μL of CJM in a 200‐μL well to achieve an inoculation level of 2 log of CFU/mL. The plate was incubated at 30°C for 96 h (4 days). The optical density at *λ*_630_ was measured for each well every hour to obtain bacterial growth curves and determine the exponential and stationary phases of growth in CJM. The data from log phase were used to calculate generation times.

### 2.2 CO_2_ production by *Enterobacteriaceae* in CJM

CO_2_ production by *Enterobacteriaceae* was evaluated in CJM contained in vacutainers so that the increasing concentrations of the gas in the medium could be measured accurately. The inocula were prepared as described above and were suspended in the same fresh CJM used for the first experiment. Each culture was diluted with CJM as needed to inoculate the experimental and sterile CJM in vacutainers to 2.0 ± 0.4 log of CFU/mL.

**FIGURE 1:**
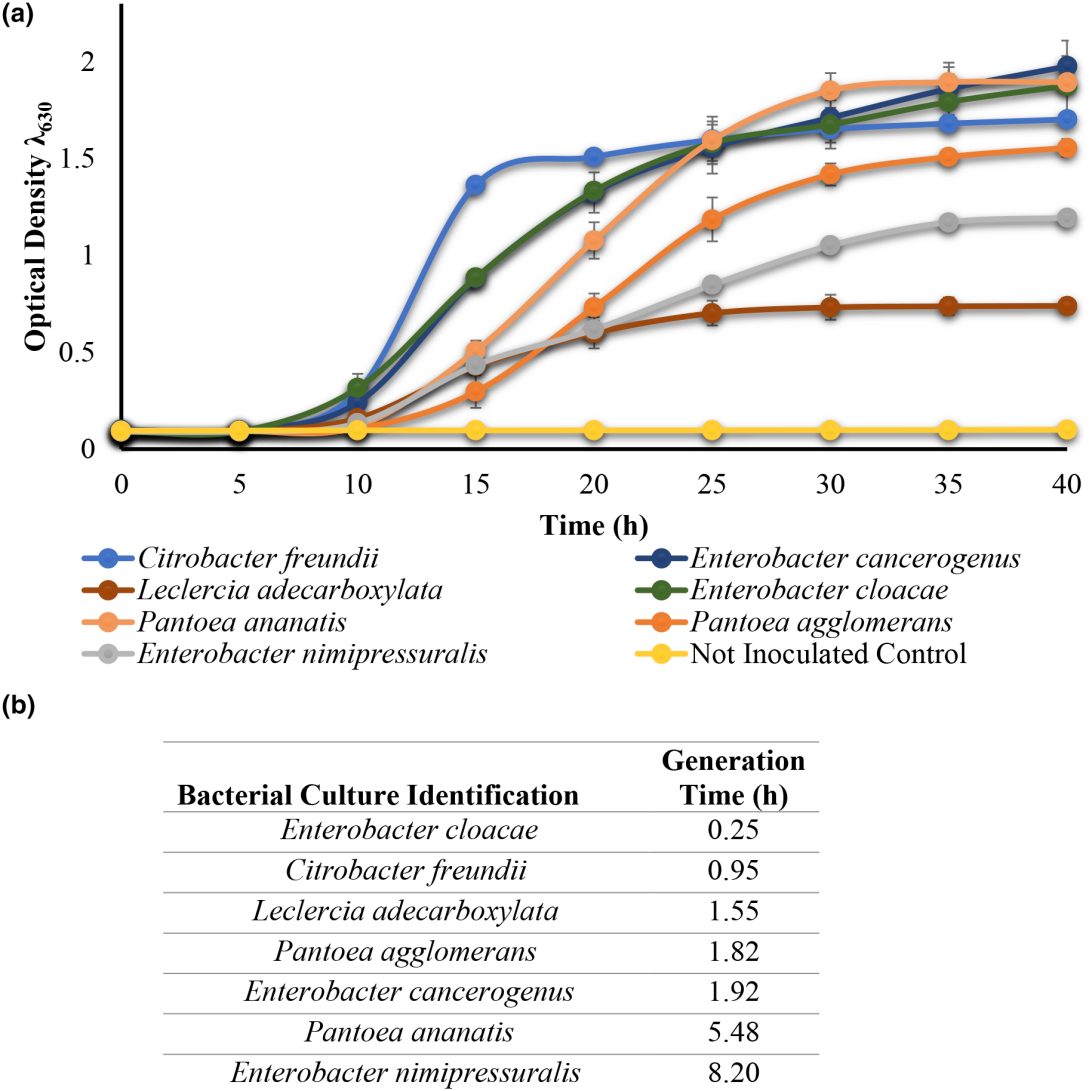
Growth of *Enterobacteriaceae* in CJM prepared from blended size 3B (1.75–2.0 in. diameter) cucumbers at 30°C in a 96‐well plate (panel a). The data shown are the average and standard deviation of duplicate samples collected from CJM prepared with one cucumber lot. The generation times for *Enterobacteriaceae* cultures in CJM are shown in (panel b).

Aliquots of 4 mL of sterile CJM were injected into 10‐mL sterile vacutainers (BD, Franklin Lakes, NJ, USA) using aseptic techniques. The vacutainers were incubated at 30°C for 48 h after inoculation. The incubation length and sampling times of 11, 18, 26, 36, and 48 h were selected based on the growth curves shown in Figure 1. Samples of 1 mL were collected from each culture through the Hemogard closure of the vacutainer (Pulmolab, 10 mL, BD #366643, Northridge, CA, USA) using a 3‐mL sterile syringe equipped with a luer‐lok tip and BD precision glide needle (BD, Franklin Lakes, NJ, USA) after incubation. The pH of culture supernatants prepared by centrifugation at 15,294 *g* for 5 min (Brushless Microcentrifuge, Denville 260D, Denville Scientific, Inc., Holliston, MA, USA) was measured with an Accumet pH meter (cat. 13‐636‐AR25B, Accumet™ AR25 pH/mV/°C/ISE, probe cat. 13–620‐290, Fisher Scientific™, Hampton, NH, USA; slope >95%). Supernatants were stored at −20°C until HPLC analysis was conducted as described below. An additional 1‐mL sample was collected from each vacutainer for determining colony counts on BHI agar plates (data not shown). The amount of CO_2_ formed in each vacutainer was measured from the remaining 2 mL of culture in each vacutainer.

An aliquot of 3 mL of a 3.33 M (20%) acetic acid solution was added to each vacutainer containing 2 mL of culture prior to vigorous shaking for 15 s to release CO_2_. The needle of a Map‐Pak Combi Gas Analyzer (AGC Instruments, Co., Clare, Ireland) was inserted through the vacutainer septa immediately after shaking to measure CO_2_ concentrations. The readings were recorded in percent (%). The CO_2_ concentrations in % were converted to the mM concentration using the conversion scale published by Zhai and Pérez‐Díaz (2017). The total number of vacutainers including duplicates with the same experimental lot of CJM for the seven cultures and one uninoculated control was 80. One vacutainer inoculated with each bacterium was sacrificed at each of the five sampling times.

Aliquots of culture supernatants were thawed and transferred into HPLC glass vials to determine the concentration of sugars, ethanol, and organic acids. The HPLC method described by McFeeters and Barish (2003) was used using an Aminex 300 × 7.8 mm HPX‐87H resin column (Bio‐Rad Laboratories, Hercules, CA, USA). The operating conditions of the system included a column temperature of 65°C and a 0.01 N sulfuric acid eluent set to flow at 0.9 mL/min. An SPD‐20A ultraviolet–visible detector (Shimadzu Corporation, Canby, OR, USA) was set at 210 nm at a rate of 1 Hz to quantify malic acid and succinic acid. A RID‐10A refractive index detector (Shimadzu Corporation) connected in series with the ultraviolet–visible detector was used to measure lactic acid, acetic acid, glucose, fructose, and ethanol. The external standardization of the detectors was done using eight gradient concentrations of the standard compounds (data not shown). The compound concentration for the samples was calculated based on the height peak of each compound in the chromatograph as compared to the corresponding compounds on the standard curves at specific retention times using the LabSolutions Workstation (Shimadzu Corporation).

The estimated limit of detection was 0.001 mM.

### 2.3 Evaluation of CO_2_ production by *Enterobacteriaceae* in a model system consisting of the fermentation of acidified and pasteurized cucumbers and evaluation of the incidence of bloater defect

Commercially available shelf‐stable Kosher dill pickles were chosen as the substrate for a model system for cucumber fermentation given that the acidification and pasteurization steps to which such product is subjected during manufacturing substantially reduce the indigenous microbial load. Three lots of shelf‐stable Kosher dills packed in 710‐mL glass jars were used for this experiment (Mt. Olive Pickle Company, Mt. Olive, NC, USA). There were two treatments and one control in the experiment that were independently triplicated by using different inocula of *E. cancerogenus* 3.2.13E or *E*. *nimipressuralis* 1.2.7E and three independent lots of Kosher dills. The processed cucumbers were alkalinized by replacing the cover liquor with a cover brine containing 20‐mM glucose, sodium hydroxide, and water to a total volume equal to that of the cover liquor volume that was originally in the jars. The replacement of the cover liquor was done using aseptic techniques. A 5 N sodium hydroxide solution (Sigma‐Aldrich, St. Louis, MO, USA) was added in the cover brine to adjust the pH of the acidified cucumbers to 6.0 ± 0.5 and enable the growth of the *Enterobacteriaceae*. The amount of sodium hydroxide needed per jar was predetermined by titrating cucumber slurries from each lot individually. The cucumber slurries consisted of 50% acidified fruits (w) and 50% of the cover brine (w) from a single experimental lot. Cucumber slurries from each experimental lot were titrated in duplicate. The volume of sodium hydroxide needed per lot was extrapolated from the titration curves and additionally adjusted based on the original cover liquor volume. The volume of the cover liquor in the commercial shelf stable pickle product varied between 299 and 338 mL. The acidified cucumbers that were covered with 299–306 g and 307–338 g of cover brine were alkalinized with 18 mL and 17 mL of 5 N sodium hydroxide, respectively. The alkaline cover brine was vacuum filtered through a 0.2‐μm membrane (Nalgene®‐Rapid Flow™, Thermo Scientific) for sterilization prior to adding it to the experimental jars. The nine jars containing the alkalinizing cucumbers and cover brine were closed with a metal lug cap with activated plastisol to achieve a tight seal. The experimental jars were then stored at 4°C for 3 days to allow for the equilibration of the cover brine components and the fruits. The jars were frequently shaken to enhance the equilibration process. The jars were opened using aseptic techniques every 24 h to collect cover brine samples for pH measurements. Cover brine samples were tempered to 21 ± 1°C for about 30 min prior to measuring pH with an Accumet pH meter. The jars were resealed as described above after each pH measurement. After 3 days of equilibration, the pH reached 6.5 ± 0.5 and the fruits were transferred to sterilized 3.9 L jars applying aseptic techniques. The metal lug caps used on the 3.9 L jars were equipped with two rubber septa to enable the insertion of Gastec ammonia and hydrogen detection tubes (catalog no. 3HM and 30, respectively). The combined cucumbers and cover brine volume of about 700 mL occupied a minor fraction of the total volume in the 3.9 L jars (about 18%) leaving a large headspace that served as a source of oxygen for the aerobic *Enterobacteriaceae*, thus promoting aerobic growth. Three jars, one from each lot, were inoculated with *E. cancerogenus* 3.2.13E. Another three jars, one from each lot, were inoculated with *E*. *nimipressuralis* 1.2.7E. The remaining three jars remained uninoculated and were used as control for the background microbial activity. The inocula were prepared as described above and added to 2 log of CFU/mL in the jars.

Changes in the gas composition in the headspace of the jars were monitored during incubation and at the endpoint. The concentrations of CO_2_ and oxygen (O_2_) in the headspace of the jars were measured at 0, 20, 24, 27, 30, 48, 71, and 74 h by inserting the sampling needle of the Map‐Pak Combi Gas Analyzer (AGC Instruments, Co.) through the rubber septa installed on the jar lid and initiating the detection on the instrument. The formation of ammonia and H_2_ was monitored at the endpoint (74 h) only, using a Gastec GV‐100S pump (Gastec Corporation) attached to ammonia or H_2_ detector tubes (Gastec Corporation). Cover brine samples were aseptically collected at the endpoint (74 h) for HPLC analysis and pH measurements were conducted as described above. The acidified and fermented cucumbers were collected for assessment of bloater defect conducted as described by Zhai and Pérez‐Díaz (2017).

Aseptically collected cover brine samples were serially diluted in 0.85% NaCl solution upon collection for spiral plating. *Enterobacteriaceae* were enumerated in Violet, Red Bile agar supplemented with 1% glucose (VRBG), which were incubated at 37°C for 24 h prior to the enumeration of all colony types. Colony counts for presumptive LAB were determined by plating on Lactobacilli deMan, Rogosa, and Sharpe (MRS) agar supplemented with 10 mL/L of a 0.1% solution of cycloheximide (SRO222C, Oxoid Ltd, Basingstoke, Hants, England) to exclude the aerobic growth of yeasts and molds. Yeasts and molds were enumerated in Yeast and Mold agar (YMA) supplemented with 0.04% chloramphenicol and 0.04% chlortetracycline to inhibit bacteria. The MRS and YMA plates were incubated at 30°C for 48 h prior to enumeration. Spiral plating was done using an Eddy Jet 2 W spiral plater (IUL Instruments, Barcelona, Spain). Colonies were enumerated using a Flash & Go Automated Colony Counter (Neutec Group, Inc., Barcelona, Spain).

The possible germination of *Bacillus* spores in the cucumber fermentation model system was monitored by plating aseptically collected cover brine samples at the 36 h time point onto MRS as described above. Eleven colonies were picked from the MRS agar plates for purification. Each purified colony was transferred to MRS broth supplemented with 15% glycerol (v/v) (Cat No. G5516, Sigma Aldrich) for the preparation of frozen stocks. Purified cultures were transferred from frozen stocks to 1 mL of MRS broth, individually, and incubated at 30°C ± 2 for 48–72 h under static aerobic conditions to obtain bacterial pellets for DNA extraction. Total genomic bacterial DNA was obtained using an InstaGene Matrix DNA extraction kit (Bio‐Rad Laboratories) following the manufacturer's instructions. Extracted DNA was used for the partial amplification of the 16S rDNA gene for the purpose of sequencing and identification. The polymerase chain reaction (PCR) amplification mixture contained 2× master mix (Bio‐Rad), 1 μL of the resulting total genomic DNA extracted from 1 out of the 11 bacterial isolates, and 0.25 μM of primers 8f (5‐AGAGTTTGATCCTGGCTCAG‐3′) and 1492r (5’‐GGTTACCTTGTTACGACTT‐3′) (Wilson et al., 1990). The PCR amplification steps consisted of 1 cycle of 4 min at 94°C followed by 25 cycles of 1 min at 94°C, 2 min at 55°C, and 2 min at 72°C, with a final extension step of 7 min at 72°C. The amplicons were stored at 4°C until sequenced by Eton Bioscience Inc. (Durham, NC, USA). Sequence data were formatted and analyzed using the BioEdit software (www.mbio.ncsu.edu/bioedit). Only bases that had quality scores greater than or equal to 20 were used for the alignment. The sequences obtained were subjected to the basic local alignment search tool (BLAST) (Altschul et al., 1990; Benson et al., 1997) using the nonredundant nucleotide database to determine the identity of the isolates. Sequences can be accessed via the National Center for Biotechnology Information website with accession numbers: MT416150 to MT416160.

### 2.4 Statistical analysis

Significant differences among the fermentation treatments were determined by LSMeans Tukey's HSD using JMP Pro 12 (SAS Institute, Inc., Cary, NC, USA). A difference between treatments based on date was considered and the interactions between treatments and sampling times were assessed. For all data sets, means marked by different letters denote statistically significant difference at a *p* ≤ .05 (ANOVA).

## 3 RESULTS

### 3.1 Growth of *Enterobacteriaceae* in CJM and CO_2_ production

*E. cloacae*, *E. cancerogenus*, *P. ananatis*, and *Citrobacter freundii* presented the highest optical density through the entire incubation period reaching a *λ*_630_ of about 2.0, equivalent to 9 log of CFU/mL as determined by plating on BHI (Figure 1). The remaining three cultures, including *P. agglomerans*, *E. nimipressuralis*, and *L. adecarboxylata* remained at a maximum cell density at *λ*_630_ of 1.5 ± 0.3 or below, which was equivalent to 6–8 log of CFU/mL (Figure 1). The calculated generation times varied between 0.25 and 8.20 h (Figure 1).

*E. cancerogenus* and *E. cloacae* produced the highest amounts of CO_2_ in CJM at 171.58 ± 42.96 and 170.89 ± 0.83 mM, respectively, after 48 h of incubation. Such amounts were 1.6‐ to 1.8‐fold that produced by *P. ananatis* (104.44 ± 0.28 mM) and *P. agglomerans* (93.33 ± 16.54 mM) and almost eight times the amount produced by the other three cultures scrutinized, including *E. nimipressuralis*, *L. adecarboxylata*, and *C. freundii* (Figure 1).

The pH of the CJM contained in vacutainers inoculated with six out of the seven isolates tested was significantly lower than that of the noninoculated control after 48 h of incubation (Table 1). The mild CJM acidification may be the result of the formation of 0‐ to 7‐mM lactic acid and 0‐ to 12‐mM acetic acid (Table 2). *E. cancerogenus* and *E. cloacae* produced the most CO_2_ and utilized the most sugars after 48 h of incubation and maintained a higher average pH at 5.5 ± 0.3 (Table 2). The *E. cancerogenus* and *E. cloacae* were unique in producing ethanol and using all the glucose initially present in the CJM (Table 2). Most bacteria utilized from 5‐ to 10‐mM malic acid from the CJM, but *E. nimipressuralis* and *L. adecarboxylata* produced 10–15 mM (Table 2). Minimal amounts of fructose and succinic acid were utilized and produced, respectively, by *Enterobacteriaceae* (Table 2). *E. cancerogenus*, *E. cloacae*, *P. ananatis*, and *P. agglomerans* produced from 10‐ to 15‐mM succinic acid (Table 2).

**TABLE 1:** CO_2_ production by *Enterobacteriaceae* in CJM and cultures endpoint pH.

Inocula	Estimated mM of CO_2_ produced in the cover brine	Endpoint fermentation pH
None (positive control)	7.22 ± 0.14^A^	5.9 ± 0.4^A^
*Enterobacter cancerogenus*	171.58 ± 42.96^B^	5.5 ± 0.2^B^
*Enterobacter cloacae*	170.89 ± 0.83^B^	5.5 ± 0.3^B^
*Pantoea ananatis*	104.44 ± 0.28^C^	5.2 ± 0.1^B^
*Pantoea agglomerans*	93.33 ± 16.54^C^	5.8 ± 0.1^AB^
*Enterobacter nimipressuralis*	16.85 ± 6.53^D^	4.86 ± 0.28^B^
*Leclercia adecarboxylata*	25.01 ± 26.69^D^	5.04 ± 0.26^B^
*Citrobacter freundii*	31.10 ± 15.57^D^	4.9 ± 0.1^B^

*Note*: The statistical analysis was performed individually for each measurement (by column). Average and standard deviation of technical duplicates with the same experimental lot of cucumber juice are shown. Levels not connected by the same letter within a column are significantly different.

**TABLE 2:** Metabolites in CJM inoculated with *Enterobacteriaceae*

Inocula	Remaining malic acid (mM)	Fermentation substrates utilized (mM)		Metabolic products (mM)			
		Glucose	Fructose	Lactic acid	Acetic acid	Succinic acid	Ethanol
None (positive control)	14.6 ± 0.9	None	None	1.0 ± 1.4	BDL	BDL	BDL
*Citrobacter freundii*	9.0 ± 8.5^A^	15.0 ± 15.5^A^	7.0 ± 5.2^A^	7.1 ± 1.34^A^	12.4 ± 6.4^A^	10.0 ± 11.5^A^	BDL
*Enterobacter cancerogenus*	2.8 ± 1.34^B^	25.1 ± 0^C^	25.5 ± 13.1^A^	BDL	BDL	13.2 ± 2.2^A^	22.0 ± 0.5^A^
*Enterobacter cloacae*	4.8 ± 2.6^A^	24.4 ± 2.5^C^	18.4 ± 3.2^A^	0.5 ± 0.7^A^	3.0 ± 1.4^A^	12.4 ± 3.4^A^	21.8 ± 0^A^
*Pantoea ananatis*	3.6 ± 0.5^B^	30.3 ± 9.7^B^	15.0 ± 5.0^A^	0.67 ± 1.0^A^	BDL	14.5 ± 0.1^A^	BDL
*Pantoea agglomerans*	7.6 ± 0.3^A^	23.7 ± 9.1^A^	11.7 ± 4.0^A^	5.1 ± 2.4^A^	1.0 ± 1.4^A^	7.8 ± 3.4^A^	BDL
*Enterobacter nimipressuralis*	26.6 ± 3.9^C^	13.8 ± 10.2^A^	10.2 ± 1.4^A^	4.2 ± 4.4^A^	6.1 ± 4.7^A^	1.0 ± 1.3^A^	BDL
*Leclercia adecarboxylata*	33.2 ± 16.5^C^	11.2 ± 16.1^A^	5.7 ± 7.6^A^	7.4 ± 6.2^A^	8.4 ± 4.6^A^	1.2 ± 1.6^A^	BDL

*Note*: The statistical analysis was performed individually for each metabolite (by column). The cultures were incubated in vacutainers at 30°C for 48 h. The data presented represent the average and standard deviation of technical duplicates with the same experimental lot of cucumber juice prepared from size 3B (1.25–1.5″ diameter) cucumbers. Levels not connected by the same letter are significantly different. Malic acid, glucose, and fructose concentrations were at 14.6 mM, 25.1 mM, and 30.3 mM, respectively, in the fresh CJM.

Abbreviation: BDL, Below Detection Level (>0.001 mM).

### 3.2 CO_2_ production by *Enterobacteriaceae* in the acidified and pasteurized cucumber fermentation model system and incidence of bloater defect

**FIGURE 2:**
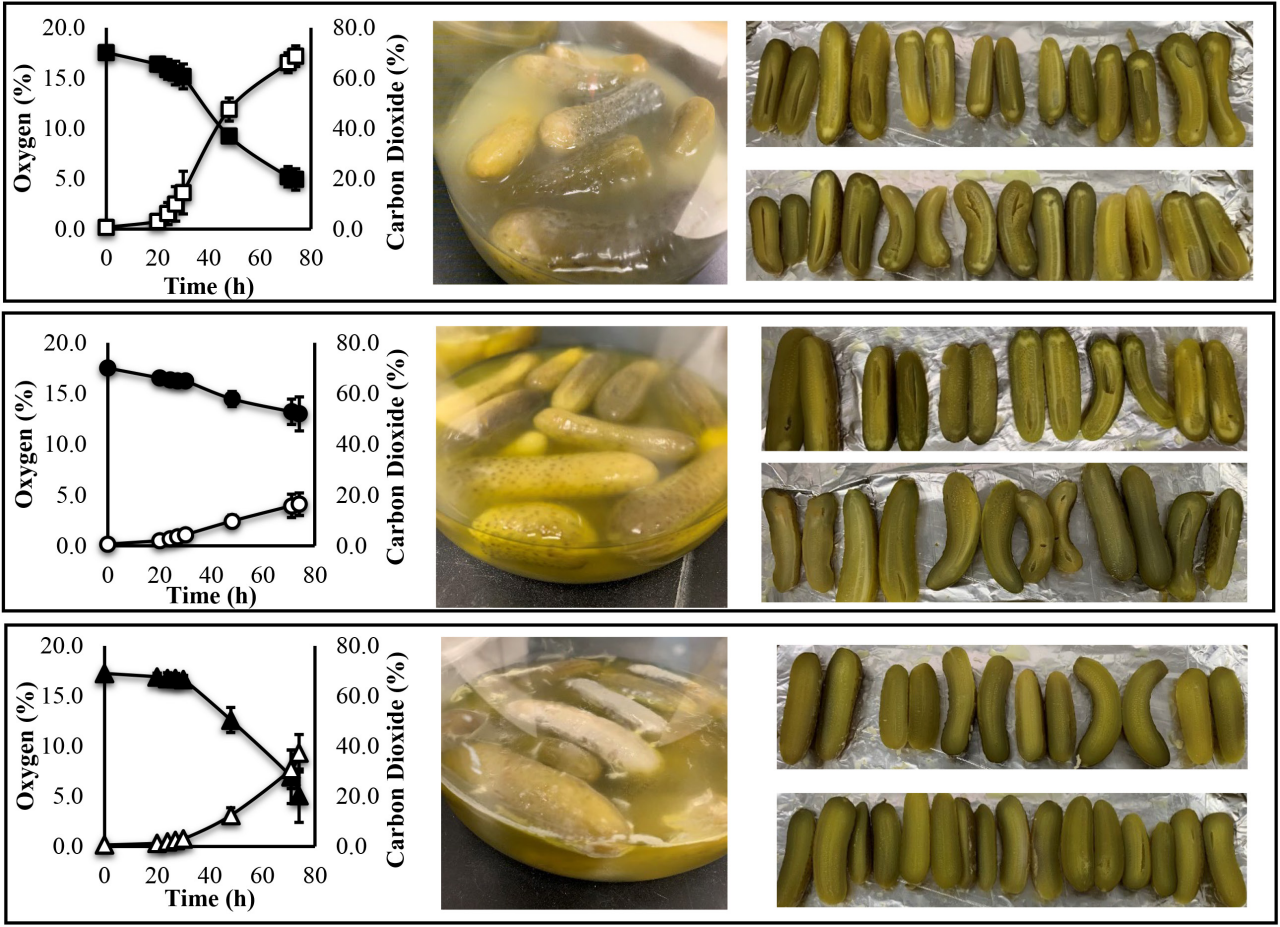
Oxygen and CO_2_ measured in the acidified fermentation jar headspace followed by images of the jars after 76 h of incubation and sliced cucumbers recovered from the jars to assess bloater defect. The first, middle, and bottom rows show the data corresponding to the jars inoculated with *E. cancerogenus*, *E. nimipressuralis*, and uninoculated control jars, respectively.

Changes in pH of the cover brine between 1 and 2 units were observed in the jars inoculated with *E. cancerogenus* and *E. nimipressuralis* (Table 3). A pH change of about 1 unit was also observed in the uninoculated control jar, which also developed a thick layer of bacterial growth at the interface of the headspace and the cover brine (Table 3 and Figure 2). Colony counts for presumptive lactobacilli reached 8.3 ± 0.7 log CFU/mL in the two treatments and control jars (Table 3). Yeast and mold growth was not observed in the two treatments or control jars (Table 3). Colony counts for *Enterobacteriaceae* from VRBG plates remained below detection levels in the uninoculated control jars and increased to 8.8 ± 0.1 and 9.3 ± 0.1 log of CFU/mL in the jars inoculated with *E. nimipressuralis* and *E. cancerogenus*, respectively (Table 3). Growth of the *Enterobacteriaceae* resulted in the production of up to 2% hydrogen, and 27 (16%) and 138 mM (69%) CO_2_ (Table 3). Although CO_2_ was also produced in the uninoculated control, there was no indication of bloater defect in the fruits collected from such jars (Table 3 and Figure 2). A bloater index of 17.42 and 25.4 was calculated for the *E. nimipressuralis* and *E. cancerogenus* treatments, respectively (Table 3). While a substantial utilization of glucose and fructose was observed in the jars inoculated with *E. cancerogenus*, nominal utilization of the sugars was detected in the jars inoculated with *E. nimipresuralis* and the uninoculated control (Table 3). Growth of *E. cancerogenus* in the brined cucumber jars produced 19.1 ± 1.0 mM ethanol and some succinic acid but no lactic acid (Table 3). Growth of *E. nimipressuralis* and that of unknown bacteria in the uninoculated control generated 13.03 ± 3.9 and 14.52 ± 3.15 mM lactic acid, respectively (Table 3). Colonies isolated from the noninoculated control jars were identified as *Bacillus tequilensis* (accession no. MT416150), *Lysinibacillus fusiformis* (accession no. MT416152 and MT416156), *Bacillus subtilis* (accession no. MT416159), and *Bacillus* sp. (accession no. MT416151, MT416153, MT416154, MT416155, MT416157, MT416158, and MT416160) by the partial sequencing of the 16S rDNA. Bacterial growth in all jars resulted in the consumption of oxygen and the production of CO_2_ (Figure 2). Growth of *E. cancerogenus* in the jars of brined cucumbers resulted in the greatest consumption of oxygen and the highest production of CO_2_ (Figure 2).

**TABLE 3:** Changes in colony counts, pH, cover brine biochemistry, and bloater index resulting from the growth of *E. cancerogenus* and *E. nimipressuralis* in brined cucumbers that were subjected to acidification and pasteurization prior to inoculation. A significant difference between a treatment and control is indicated by superscript letters.

Measurements	Noninoculated control	*Enterobacter nimipressuralis*	*Enterobacter cancerogenus*
pH measurements			
Initial equilibrated pH	6.47 ± 0.44^A^	6.79 ± 0.59 ^A^	6.78 ± 0.50 ^A^
Endpoint pH	5.24 ± 0.16^A^	4.88 ± 0.08^B^	5.95 ± 0.12^C^
Change in pH	1.23	1.91	0.83
Colony counts from Agar Plates (log of CFU/mL)			
VRBG	0^A^	8.8 ± 0.1^B^	9.3 ± 0.1^B^
Lactobacilli MRS	8.0 ± 0.4^A^	8.2 ± 0.7^A^	8.6 ± 1.0^A^
YMA	No growth	No growth	No growth
Amount of gases produced (%) and bloater index			
Hydrogen	BDL	2	2
Ammonia	BDL	BDL	BDL
Carbon dioxide	37.2 ± 7.5^A^	16.4 ± 4.5^B^	68.7 ± 4.1^C^
Bloater index	0.7 ^A^	17.42 ^B^	25.4 ^B^
Biochemical composition of the cover brine (mM)			
Glucose remaining	13.7 ± 3.3^A^	10.6 ± 2.8^A^	0^B^
Fructose remaining	19.6 ± 2.4^A^	19.7 ± 2.2^A^	1.1 ± 0.1^B^
Acetic acid remaining	62.7 ± 0.3^A^	70.5 ± 1.0^A^	51.9 ± 3.9^A^
Ethanol formed	0^A^	0^A^	19.1 ± 1.0^B^
Succinic acid formed	0^A^	4.1 ± 1.6^B^	8.2 ± 0.2^B^
Lactic acid formed	13.03 ± 3.9^A^	14.52 ± 3.15^A^	0^B^

*Note*: The statistical analysis was performed individually for each measurement (by row).

Abbreviation: BDL, Below Detection Limit.

## 4 DISCUSSION

*Enterobacteriaceae* biomass production was positively associated with CO_2_ formation in CJM. The *Enterobacteriaceae* inoculated in CJM reached maximum cell densities in 18 h. The CJM used for experimentation contained about equal concentrations of glucose and fructose at 30 ± 8 mM each and 10 ± 3 mM malic acid with a 5.9 ± 0.1, which agrees with the values documented by Lu et al. (2002). However, nominal amounts of acids, including lactic, acetic, and succinic, were produced by the *Enterobacteriaceae* in CJM, if stoichiometry is considered. The lack of carbon balance could derive from the redirection of pyruvate to the synthesis of amino acid, fatty acids, and other biologically relevant molecules and/or the utilization of the respiratory pathway. Aerobic growth supported by the citric acid cycle is described for *Enterobacteriaceae*, particularly *Enterobacter aerogenes* (Antranikian & Giffhorn, 1987). The production of ethanol by the two species that generated the highest amounts of CO_2_ suggests the utilization of pyruvate catabolism to regenerate NADH and/or produce ATP by substrate‐level phosphorylation via acetyl‐phosphate (Rothwell et al., 2022).

The production of malic acid by *E. nimipressuralis* and *L. adecarboxylata* to 12.0 ± 1.9 and 18.6 ± 12.2 mM, respectively, suggests a different mode of TCA cycle utilization relative to other *Enterobacteriaceae*. Secretion of malic acid by fungi represents an energy‐deriving metabolic pathway, particularly for *Aspergillus oryzae* (Knuf et al., 2013). *A. oryzae* uncouples growth and malic acid production under stationary phase by utilizing the reductive cytosolic TCA branch from pyruvate. Engineered *E. coli* are also capable of secreting L‐malic acid (Chi et al., 2016; West, 2017). This strategy facilitates the consumption of glucose making it unavailable for the competing microbes (Knuf et al., 2013). The possible production of malic acid by such bacteria in a cucumber fermentation could exacerbate bloater defect by increasing the amounts of CO_2_ that could be formed from the decarboxylation of the organic acid by the indigenous microbiota.

A cucumber fermentation model system, composed of acidified cucumbers that were commercially pasteurized, was used in this study to confirm that the sole metabolic activity of *Enterobacteriaceae* could induce bloater defect. Although most of the indigenous microbiota was inactivated in this model system prior to the adjustment in pH to 6.5 ± 0.5, several bacilli were isolated from the uninoculated control jars. *Bacillus tequilensis*, *Lysinibacillus fusiformis*, *Bacillus subtilis*, and *Bacillus mobilis* were isolated from the control jars and are known to be aerobic or facultative anaerobic, spore formers that are ubiquitous in the environment and foods (Gatson et al., 2006; Liu et al., 2017; Logan & De Vos, 2015). It is speculated that the spores that survived the pasteurization and acidification steps germinated in the control jars that contained 20 mM glucose and an aerobic headspace. Of interest was the observation that the confluent growth in the control jars was initiated on the interface of the headspace and cover brine or cucumbers (Figure 2). The subsurface brine remained translucent for the first 48 h of experimentation. While CO_2_ was produced in the control jars, bloater defect was not observed. It is presumed that the production of CO_2_ was associated with superficial bacterial growth and that the gas produced was mainly distributed in the headspace. Inoculation of the model system with *E. cancerogenus* resulted in colony counts of 9.3 ± 0.1 log of CFU/mL on VRBG plates which contrasted with below detection levels from the noninoculated control jars. CO_2_ was produced to 137 mM (69 ± 4%) in the jars inoculated with *E. cancerogenus*, almost 2‐fold of that produced in the control jars (37.2 ± 7.5%), which generated a bloater index of 25.4. Growth of *E. nimipressuralis* was less abundant than that observed by *E. cancerogenus* and the metabolic footprint generated by such bacterium was in line with that observed in CJM characterized by delayed sugar utilization, production of lactic acid, and a greater decrease in pH. CO_2_ production was observed at 35 mM (16 ± 5%) in the jars inoculated with *E. nimipressuralis*, lower than that produced in the uninoculated control jars, which generated a bloater index of 17.42. Different from the control jars, the inoculation of *Enterobacteriaceae* in the model system generated detectable concentrations of H_2_ (2%), a metabolic activity that has been associated with *Enterobacter* sp. (Etchells et al., 1968). A reduction in the amount of oxygen present in the headspace of the jars was observed from both treatments and the control jars. These observations suggest that the propagation of *Enterobacteriaceae* in the acidified and pasteurized cucumbers prevented the germination of *Bacillus* spores, as seen on the control jars, and that the metabolic activity of *Enterobacteriaceae*, distributed within the fermentation substrate, was sufficient to cause bloater defect. The utilization of oxygen from the headspace further confirms the use of a respiratory pathway by the bacilli and enterobacilli.

## 5 CONCLUSION

*Enterobacteriaceae* are identified as agents capable of producing sufficient CO_2_ in cucumber fermentation to induce bloater defect. Such organisms could produce CO_2_, H_2_, malic acid, succinic acid, and ethanol in cucumber fermentation. We previously documented that the ability of *Enterobacteriaceae* to proliferate in cucumber fermentation is impaired by the cover brine constituents and the indigenous microbiota. The data presented here suggest that the indigenous *Enterobacteriaceae* can induce the CO_2_‐mediated bloater defect if the fermentation conditions permit their growth. Thus, it is necessary to ensure that fermentation conditions are inhibitory of *Enterobacteriaceae* in the early stage of cucumber fermentation to preserve quality.

Given that *Leuconostocaceae* can also induce the CO_2_‐mediated bloater defect in cucumber fermentation, the coexistence of such lactic acid bacteria with *Enterobacteriaceae* in indigenous cucumber fermentations, particularly those brined with low salt, could lead to the accumulation of gases in levels that are sufficient to cause the defect or aggravate it. It is presumed that the main contribution to CO_2_ production by both bacterial families comes from the heterofermentation of the primary sugars in cucumber, glucose, and fructose. Because both bacterial families, *Enterobacteriaceae* and *Leuconostocaceae*, prevail in commercial cucumber fermentation up to day 3, strategies to inhibit their metabolism early in the process are expected to ameliorate the incidence of bloater defect. The fundamental knowledge generated by this study enables the development of strategies to consistently prevent bloater defect in cucumber fermentation.

## AUTHOR CONTRIBUTIONS

**Yawen Zhai:** Data curation (equal); formal analysis (equal); investigation (equal); methodology (equal); validation (equal); visualization (equal); writing – original draft (equal); writing – review and editing (equal). **Christian G. Pagán‐Medina:** Data curation (equal); investigation (equal); methodology (equal); validation (equal); visualization (equal); writing – review and editing (equal). **Ilenys Muniz Pérez‐Díaz:** Conceptualization (lead); data curation (equal); formal analysis (equal); funding acquisition (lead); investigation (equal); methodology (equal); project administration (lead); supervision (lead); visualization (equal); writing – original draft (equal); writing – review and editing (equal).

## FUNDING INFORMATION

The research presented here was conducted as part of the United States Department of Agriculture, Agricultural Research Service national project plan no. 6070–41,000‐008‐00D.

## CONFLICT OF INTEREST STATEMENT

The authors declare no conflict of interest.

## Data Availability

Data are shown in the article.

## References

[fsn33557-bib-0001] Altschul, S. F. , Gish, W. , Miller, W. , Meyers, E. W. , & Lipman, D. J. (1990). Basic local alignment search tool. Journal of Molecular Biology, 215, 403–410. 10.1016/S0022-2836(05)80360-2 2231712

[fsn33557-bib-0002] Antranikian, G. , & Giffhorn, F. (1987). Citrate metabolism in anaerobic bacteria. FEMS Microbiological Letters, 46(2), 175–198. 10.1016/0378-1097(87)90063-2

[fsn33557-bib-0003] Benson, D. A. , Boguski, M. S. , Lipman, D. J. , & Ostell, J. (1997). GenBank. Nucleic Acids Research, 25, 1–6. 10.1093/nar/gkv1276 9016491 PMC146400

[fsn33557-bib-0004] Chi, Z. , Wang, Z. , Wang, G. , Khan, I. , & Chi, Z. (2016). Microbial biosynthesis and secretion of L‐malic acid and its applications. Critical Reviews in Biotechnology, 36(1), 99–107. 10.3109/07388551.2014.924474 25025277

[fsn33557-bib-0005] Ciani, M. , Comitini, F. , & Mannazzu, I. (2008). Fermentation. In B. Fath (Ed.), Encyclopedia of ecology (pp. 310–321). Elsevier. 10.1016/B978-0-12-409548-9.00693-X

[fsn33557-bib-0006] Corey, K. A. , Pharr, D. M. , & Fleming, H. P. (1983). Role of the osmoticum in bloater formation of pickling cucumbers. Journal of Food Science, 48(1), 197–201. 10.1111/j.1365-2621.1983.tb14822.x

[fsn33557-bib-0007] Etchells, J. L. (1941). Incidence of yeasts in cucumber fermentations. Food Research, 6(1), 95–104. 10.1111/j.1365-2621.1941.tb16272.x

[fsn33557-bib-0008] Etchells, J. L. , Bell, T. A. , Fleming, H. P. , Kelling, R. E. , & Thompson, R. L. (1974). Advisory statement on Q‐BAT instruction sheet with bloater chart. *Pickle Packers International, Inc* .

[fsn33557-bib-0010] Etchells, J. L. , Borg, A. F. , & Bell, T. A. (1968). Bloater formation by gas‐forming lactic acid bacteria in cucumber fermentations. Applied Microbiology, 16(7), 1029–1035. 10.1128/am.16.7.1029-1035.1968 16349808 PMC547583

[fsn33557-bib-0011] Etchells, J. L. , Fabian, F. W. , & Jones, I. D. (1945). The *Aerobacter* fermentation of cucumbers during salting. Michigan Agricultural Experiment Station Technical Bulletin, 200, 56.

[fsn33557-bib-0012] Etchells, J. L. , & Jones, I. D. (1941). An occurrence of bloaters during the finishing of sweet pickles. Fruit Production Journal, 20(12), 370–381.

[fsn33557-bib-0014] Fleming, H. P. , & Pharr, D. M. (1980). Mechanism for bloater formation in brined cucumbers. Journal of Food Science, 45(6), 1595–1600. 10.1111/j.1365-2621.1980.tb07570.x

[fsn33557-bib-0015] Fleming, H. P. , Thompson, R. L. , Etchells, J. L. , Kelling, R. E. , & Bell, T. A. (1973a). Bloater formation in brined cucumbers fermented by *lactobacillus plantarum* . Journal of Food Science, 38, 499–503. 10.1111/j.1365-2621.1973.tb01466.x

[fsn33557-bib-0016] Fleming, H. P. , Thompson, R. L. , Etchells, J. L. , Kelling, R. E. , & Bell, T. A. (1973b). Carbon dioxide production in the fermentation of brined cucumbers. Journal of Food Science, 38, 504–506. 10.1111/j.1365-2621.1973.tb01467.x

[fsn33557-bib-0019] Gatson, J. W. , Benz, B. F. , Chandrasekaran, C. , Satomi, M. , Venkateswaran, K. , & Hart, M. E. (2006). *Bacillus tequilensis* sp. *nov*., isolated from a 2000‐year‐old Mexican shaft‐tomb, is closely related to *bacillus subtilus* . International Journal of Systematic and Evolutionary Microbiology, 56(7), 475–1484. 10.1099/ijs.0.63946-0 16825615

[fsn33557-bib-0043] Iversen, C. (1994). Enterobacter. In R. K. Robinson , C. A. Batt , & P. D. Patel (Eds.), Encyclopedia of food microbiology (pp. 653–666). Elsevier Science and Technology.

[fsn33557-bib-0021] Knuf, C. , Nookaew, I. , Brown, S. H. , McCulloch, M. , Berry, A. , & Nielsen, J. (2013). Investigation of malic acid production in *aspergillus oryzae* under nitrogen starvation conditions. Applied and Environmental Microbiology, 79(19), 6050–6058. 10.1128/AEM.01445-13 23892740 PMC3811345

[fsn33557-bib-0022] Liu, Y. , Du, J. , Lai, Q. , Zeng, R. , Ye, D. , Xu, J. , & Shao, Z. (2017). Proposal of nine novel species of the *Bacillus cereus* group. International Journal of Systematic and Evolutionary Microbiology, 67(8), 2499–2508. 10.1099/ijsem.0.001821 28792367

[fsn33557-bib-0023] Logan, N. A. , & De Vos, P. (2015). Bacillus. In Bergeys' manual of systematics of archaea and bacteria. John Wiley & Sons, Inc. & Bergey's Manual Trust. 10.1002/9781118960608.gbm00530

[fsn33557-bib-0024] Lu, Z. , Fleming, H. P. , & McFeeters, R. F. (2002). Effects of fruit size on fresh cucumber composition and the chemical and physical consequences of fermentation. Journal of Food Science, 67(8), 2934–2939. 10.1111/j.1365-2621.2002.tb08841.x

[fsn33557-bib-0025] McDonald, L. C. , Fleming, H. P. , & Daeschel, M. A. (1991). Acidification effects on microbial populations during initiation of cucumber fermentation. Journal of Food Science, 56(5), 1353–1356. 10.1111/j.1365-2621.1991.tb04771.x

[fsn33557-bib-0026] McFeeters, R. F. , & Barish, A. O. (2003). Sulfite analysis of fruits and vegetables by high‐performance liquid chromatography (HPLC) with ultraviolet spectrophotometric detection. Journal of Agriculture and Food Chemistry, 51(6), 1513–1517. 10.1021/jf025693c 12617575

[fsn33557-bib-0028] Passos, F. V. , Felder, R. M. , Fleming, H. P. , McFeeters, R. F. , & Ollis, D. F. (2005). Dynamic model for mass transfer of solutes in cucumber fermentation. Journal of Food Engineering, 68(3), 297–302. 10.1016/j.jfoodeng.2004.06.002

[fsn33557-bib-0029] Pérez‐Díaz, I. M. , Hayes, J. S. , Medina, E. , Webber, A. M. , Butz, N. , Dickey, A. N. , Lu, Z. , & Azcarate‐Peril, M. A. (2018). Assessment of the non‐lactic acid bacteria microbiota in fresh cucumbers and commercially fermented cucumber pickles brined with 6% NaCl. Food Microbiology, 77, 202–207. 10.1016/j.fm.2018.08003 30297040

[fsn33557-bib-0031] Pérez‐Díaz, I. M. , McFeeters, R. F. , Moeller, L. , Johanningsmeier, S. D. , Hayes, J. S. , Fornea, D. , Gilbert, C. , Custis, N. , Beene, K. , & Bass, D. (2015). Commercial scale cucumber fermentations brined with calcium chloride instead of sodium chloride. Journal of Food Science, 80(12), M2827–M2836. 10.1111/1750-3841.13107 26512798

[fsn33557-bib-0032] Rothwell, M. A. R. , Zhai, Y. , Pagan‐Medina, C. G. , & Pérez‐Díaz, I. M. (2022). Growth of ɣ‐proteobacteria in low salt cucumber fermentation is prevented by lactobacilli and the cover brine ingredients. In Growth of ɣ‐proteobacteria in low salt cucumber fermentations is prevented by lactobacilli and the cover brine ingredients. Microbiology Spectrum. Advance online publication. 10.1128/spectrum.01031-21 PMC9241618 35543556

[fsn33557-bib-0035] Unden, G. , & Bongaerts, J. (1997). Alternative respiratory pathways of *Escherichia coli*: Energetics and transcriptional regulation in response to electron acceptors. Biochimica et Biophysica Acta, 1320(3), 217–234. 10.1016/s0005-2728(97)00034-0 9230919

[fsn33557-bib-0036] Veldhuis, M. K. , & Etchells, J. L. (1939). Gaseous products of cucumber pickle fermentations. Food Research, 4(6), 621–630. 10.1111/j.1365-2621.1939.tb17159.x

[fsn33557-bib-0037] West, T. P. (2017). Microbial production of malic acid from biofuel‐related coproducts and biomass. Fermentation, 3(2), 14. 10.3390/fermentation3020014

[fsn33557-bib-0038] Wilson, K. H. , Blitchington, R. B. , & Greene, R. C. (1990). Amplification of bacterial 16S ribosomal DNA with polymerase chain reaction. Journal of Clinical Microbiology, 28, 942–1946. 10.1128/jcm.28.9.1942-1946.1990 PMC268083 2095137

[fsn33557-bib-0040] Zhai, Y. , & Pérez‐Díaz, I. M. (2017). Fermentation cover brine reformulation for cucumber processing with low salt to reduce bloater defect. Journal of Food Science, 82(12), 2987–2996. 10.1111/1750-3841.13945 29125622

[fsn33557-bib-0041] Zhai, Y. , & Pérez‐Díaz, I. M. (2020). Contribution of *Leuconostocaceae* to CO_2_‐mediated bloater defect in cucumber fermentation. Food Microbiology, 91, 103536. 10.1016/j.fm.2020.103536 32539962

[fsn33557-bib-0042] Zhai, Y. , & Pérez‐Díaz, I. M. (2021). Identification of potential causative agents of the CO_2_‐mediated bloater defect in low salt cucumber fermentation. International Food Microbiology Journal, 16(344), 109115. 10.1016/j.ijfoodmicro.2021.109115 33662901

